# Pulsed electromagnetic fields after arthroscopic treatment for osteochondral defects of the talus: double-blind randomized controlled multicenter trial

**DOI:** 10.1186/1471-2474-10-83

**Published:** 2009-07-10

**Authors:** Christiaan JA van Bergen, Leendert Blankevoort, Rob J de Haan, Inger N Sierevelt, Duncan E Meuffels, Pieter RN d'Hooghe, Rover Krips, Geert van Damme, C Niek van Dijk

**Affiliations:** 1 Orthopaedic Research Center Amsterdam, Department of Orthopaedic Surgery, Academic Medical Center, Amsterdam, The Netherlands; 2 Department of Clinical Epidemiology and Biostatistics, Academic Medical Center, Amsterdam, The Netherlands; 3 Department of Orthopaedic Surgery, Erasmus MC, University Medical Center Rotterdam, The Netherlands; 4 Department of Orthopaedic Surgery, Stedelijk Ziekenhuis, Roeselare, Belgium; 5 Department of Orthopaedic Surgery, Diaconessenhuis, Leiden, The Netherlands; 6 Department of Orthopaedic Surgery, Algemeen Ziekenhuis Sint Lucas, Brugge, Belgium

## Abstract

**Background:**

Osteochondral talar defects usually affect athletic patients. The primary surgical treatment consists of arthroscopic debridement and microfracturing. Although this is mostly successful, early sport resumption is difficult to achieve, and it can take up to one year to obtain clinical improvement. Pulsed electromagnetic fields (PEMFs) may be effective for talar defects after arthroscopic treatment by promoting tissue healing, suppressing inflammation, and relieving pain. We hypothesize that PEMF-treatment compared to sham-treatment after arthroscopy will lead to earlier resumption of sports, and aim at 25% increase in patients that resume sports.

**Methods/Design:**

A prospective, double-blind, randomized, placebo-controlled trial (RCT) will be conducted in five centers throughout the Netherlands and Belgium. 68 patients will be randomized to either active PEMF-treatment or sham-treatment for 60 days, four hours daily. They will be followed-up for one year. The combined primary outcome measures are (a) the percentage of patients that resume and maintain sports, and (b) the time to resumption of sports, defined by the Ankle Activity Score. Secondary outcome measures include resumption of work, subjective and objective scoring systems (American Orthopaedic Foot and Ankle Society – Ankle-Hindfoot Scale, Foot Ankle Outcome Score, Numeric Rating Scales of pain and satisfaction, EuroQol-5D), and computed tomography. Time to resumption of sports will be analyzed using Kaplan-Meier curves and log-rank tests.

**Discussion:**

This trial will provide level-1 evidence on the effectiveness of PEMFs in the management of osteochondral ankle lesions after arthroscopy.

**Trial registration:**

Netherlands Trial Register (NTR1636)

## Background

Osteochondral defects (ODs) of the talus often have a severe impact on the quality of life of the patients. The patients are usually young and athletic; most are male (62%) in the third decade of their lives after a traumatic ankle sprain [1]. The primary treatment of a symptomatic OD consists of arthroscopic debridement and microfracturing [2]. This treatment yields 87% good or excellent results [3]. However, it can take up to one year to obtain improvement of clinical symptoms. Moreover, it is a great challenge to achieve early resumption of sports, which is the main goal of many of these young patients. In a series published in 2007, 26 "high-demand" athletic patients with an OD returned to sports at a mean of 15 weeks after debridement and microfracturing [4]. If we could shorten this period, we would considerably improve the quality of life in these active patients.

A potential solution to obtain this goal is the application of pulsed electromagnetic fields (PEMFs). Bassett in the 1960s and 1970s introduced and improved the clinical use of this treatment modality [5,6]. Since then, PEMFs have been applied increasingly, including their use in the treatment of osteoarthritis and (non-united) fractures [7,8]. They are designed as a portable PEMF generator, which consists of electromagnetic fields with an on-off effect of pulsing. This produces athermal effects that suppress inflammation, promote tissue healing, and relieve pain [9]. In vitro and in vivo studies have shown that PEMFs act as adenosine A2a agonists, leading to an increase of Transforming Growth Factor β-1, thereby improving bone development, reducing cartilage damage and increasing chondrocyte proliferation [10-21]. These results clearly indicate improved regeneration of bone and possibly cartilage in a scientific setting.

Clinically, its favorable effects are less obvious. PEMF as a solitary treatment for osteoarthritis of the knee has been repeatedly investigated, with conflicting results [7,22-25]. Although the effect of PEMFs on osteoarthritis of the knee seems equivocal, their value in the additional treatment of other bony and cartilaginous pathologies is promising. PEMFs have been proven as a successful method in fracture healing, especially in the case of non-union [8,26,27]. PEMF-treatment also favors the recovery of patients after arthroscopic treatment of chondral lesions in the knee, and reduces the use of non-steroidal anti-inflammatory drugs [28]. To our knowledge, sport resumption with the use of PEMFs has not been investigated. Based on the above data, we believe that PEMFs may act on ODs by improving bone regeneration and suppressing inflammation evoked by surgery.

When the above results are combined, it seems justified to state that additional PEMF-treatment may contribute to the management of ODs. Our study question is: "Does treatment with PEMFs compared to sham device lead to earlier resumption of sports in a higher percentage of patients with an osteochondral defect of the talus after arthroscopic debridement and microfracturing?".

## Methods/Design

### Study design and informed consent

The study is designed as a double-blind, randomized, placebo controlled, multicenter trial, which is in accordance with the Declaration of Helsinki [29]. The methodology will follow CONSORT (Consolidation of Standards of Reporting Trials) guidelines [30,31]. Five centers in the Netherlands and Belgium will participate. Approval has been obtained from the local Medical Ethics Committees in the participating centers (MEC 08/236). Written informed consent for participation in the study will be obtained from all patients at study entry. An information letter notifying the patients' participation will be sent to their general practitioners.

### Randomization

The participants will be randomized to receive either active PEMF-treatment or sham device, stratified for participating center, body mass index (≤/> 25 kg/m^2^) [32,33], and diameter of the defect on computed tomography (CT) (≤/> 10 mm) [1]. Randomization will be performed in randomly allocated blocks of two or four patients using ALEA, a validated web-based computer program [34]. The provider of the PEMF-devices (IGEAmedical, Carpi, Italy) will supply an equal number of active and sham devices identified by code numbers which correspond to the randomization program. Treatment allocation will be managed by an independent, unblinded research assistant (IS), who will not be involved in patient care or assessment. Patients and treating physicians as well as medical assessors will be blinded to the allocation of treatment. The code numbers will not be broken until all patients have completed the study.

### Inclusion criteria

• Patients with a symptomatic OD of the talus who are scheduled for arthroscopic debridement and microfracture [2]

• OD diameter < 15 mm on CT (in three dimensions: medial-lateral, anterior-posterior and superior-inferior)

• Ankle Activity Score (AAS) ≥ 4 before symptoms (Table 1) [35]

**Table 1:** Ankle Activity Score by Halasi et al. [35]

		Ankle Activity Score^a^		
Category	Sports and Activities	T	C	R

10	American football	10	9	8
	Basketball	10	9	8
	Gymnastics	10	9	8
	Handball	10	9	8
	Rugby	10	9	8
	Soccer	10	9	8
9	Hockey	9	8	7
	Korfball	9	8	7
	Martial arts: judo, karate, kung fu, taekwondo	9	8	7
	Orienteering	9	8	7
	Rhythmic gymnastics	9	8	7
	Volleyball	9	8	7
8	Boxing	8	7	6
	Freestyle snowboarding	8	7	6
	Ice hockey	8	7	6
	Tennis	8	7	6
	Wrestling	8	7	6
7	Aerobics, fitness	7	6	5
	Badminton	7	6	5
	Baseball	7	6	5
	Cross-country running (running on uneven ground)	7	6	5
	Modern pentathlon	7	6	5
	Squash	7	6	5
	Surfing, windsurfing	7	6	5
	Table tennis	7	6	5
	Track and field: field events	7	6	5
	Water skiing	7	6	5
6	Dancing	6	5	4
	Fencing	6	5	4
	Floorball	6	5	4
	Mountain and hill climbing	6	5	4
	Nordic skiing	6	5	4
	Parachuting	6	5	4
	Softball	6	5	4
	Special professions and working activities^b^	6		
5	Diving	5	5	4
	Scuba diving	5	5	4
	Skating, in-line skating	5	5	4
	Track and field: track events (running on even ground)	5	5	4
	Triathlon	5	5	4
	Weightlifting, body building	5	5	4
	All competitive sports of categories 4 and 3 with seasonal conditioning	5		
	Heavy physical work	5		
4	Alpine skiing and snowboarding	4	4	4
	Bowling/curling	4	4	4
	Golf	4	4	4
	Mountain biking/bmx	4	4	4
	Power lifting	4	4	4
	Sailing	4	4	4
	Physical work	4		
3	Cycling	3	3	3
	Equestrian	3	3	3
	Motorsports, technical sports	3	3	3
	Rowing, kayaking	3	3	3
	Shooting, archery	3	3	3
	Water polo and swimming	3	3	3
	Able to walk on any uneven ground	3		
2	No sports, everyday activities not limited	2		
1	Able to walk on even ground, but everyday activities limited	1		
0	Unable to walk, disabled because of ankle problems	0		

^a^T, top level (international elite, professional, national team, or first division); C, lower competitive levels; R, recreational level (participation should be considered only if it exceeds 50 hours per year).

^b^Special professions include ballet dancer, professional soldier, special rescue worker, stuntperson, and so forth.

If multiple options are applicable, the highest level is chosen.

• Age 18 years or older

### Exclusion criteria

• Concomitant OD of the tibia

• Ankle osteoarthritis grade 2 or 3 [36]

• Ankle fracture < 6 months before scheduled arthroscopy

• Surgical treatment of the index ankle performed < 1 year before scheduled arthroscopy

• Concomitant painful or disabling disease of the lower limb

• Rheumatoid arthritis

• Pregnancy

• Implanted pacemaker

• Participation in concurrent trials

• Participation in previous trials < 1 year, in which the subject has been exposed to radiation (radiographs or CT)

• Patients who are unable to fill out questionnaires and cannot have them filled out

• No informed consent

### Device description

**Figure 1:**
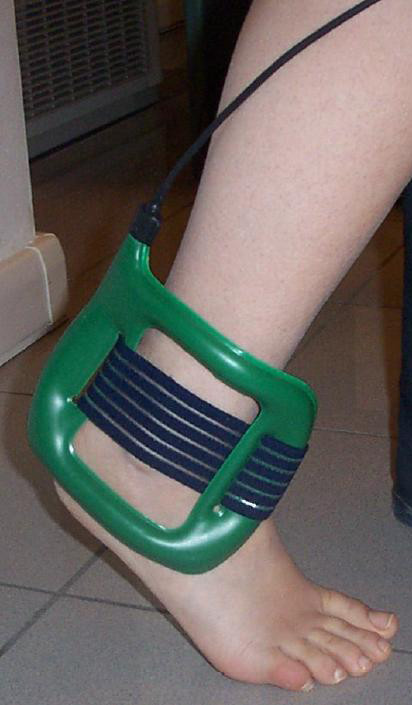
**The application of pulsed electromagnetic fields on the ankle, generated in the green coil and attached with the elastic band (I-ONE, IGEAmedical, Carpi, Italy)**.

PEMFs are applied using a portable generator attached to the ankle (Figure 1). The coil in the active treatment device generates a peak magnetic field intensity of 1.5 mT, supplied by an electric pulse frequency of 75 Hz [37]. The sham devices do not differ from active devices in shape, color, weight, and in acoustic or visual signaling. Neither the active nor the sham device produces noise or sensation and they are entirely indistinguishable. The only difference is the generated magnetic field; the sham device produces a negligible peak of less than 0.05 mT, supplied by the minimal current necessary to power the device indicators.

### Standard treatment and investigational treatment

All surgical procedures will be performed using a standardized technique [2]. Briefly, the ankle joint is approached by arthroscopy using an anterior or posterior approach. The OD is identified with a probe and debrided with a curette and bonecutter shaver. All unstable bone and cartilage are removed. After full debridement, the subchondral bone is perforated with a microfracture awl, with intervals of approximately 3 mm. At the end of the procedure a pressure bandage is applied.

After surgery the protocol-based rehabilitation program, guided by a physiotherapist, will be equal in both groups. It will be initiated with partial (eggshell) weight bearing on crutches, as tolerated, and progressed to full weight bearing over a period of six weeks. During this period active non-weight-bearing and partial weight-bearing sagittal range of motion exercises are encouraged, i.e., 15 minutes twice daily. After this six week' period, resumption of sports will be permitted as tolerated, and will not be directed by the clinician.

In both groups the investigational treatment (active PEMF-treatment or sham device treatment) will start within three days after surgery. It will be applied four hours daily (in one or two sessions) for a period of 60 days [37,38]. The patients' compliance will be monitored by a clock inside the device that records the hours of stimulation.

The prescription of nonsteroidal anti-inflammatory drugs will be avoided due to their negative effect on bone regeneration [39]. The use of paracetamol will be allowed up to a maximum dose of 4 g/d and will be discontinued one week before the visits at baseline, 1 month, 2 months, 6 months and 1 year.

### Outcome measures

The combined primary outcome measures are:

(a) the number of patients that resume and maintain sports during 12 months follow-up, and

(b) the time to resumption of sports, defined by the AAS.

Secondary outcome measures are:

- time to resumption of work,

- American Orthopaedic Foot and Ankle Society – Ankle-Hindfoot Scale (AOFAS-AHS),

- Foot and Ankle Outcome Score (FAOS),

- quality of life (EuroQol-5D),

- pain (Numeric Rating Scale),

- satisfaction (Numeric Rating Scale),

- computed tomography, and

- adverse events.

### Definitions

#### Primary outcome measures

Because there is no consensus as to defining what actual resumption of sport is – as Saxena and Eakin stated [4] – we define time to resumption of sports as the time after arthroscopy (weeks) until initiation of any sport with a minimum level of the pre-symptoms level minus 1 point on AAS, and maintained for at least 30 days. If a patient's activity level decreases to below the minimum level within 30 days after sport resumption, the resumption date will not be counted. To evaluate the level of sport activity, we will use the AAS that has been developed and validated by Halasi and associates [35]. This 10-point score is based on the type and level of sport or work, with 0 points indicating the lowest activity and 10 points indicating the highest activity (Table 1).

#### Secondary outcome measures

Resumption of work is defined as the ability to perform normal work exercises without any deficits in work quality [40]. The AOFAS-AHS is a frequently used combined objective-subjective 100-point scale which devotes 40 points to pain, 50 points to function, and 10 points to alignment [41]. The subjective part was recently validated [42]. The FAOS is a subjective 42-item questionnaire assessing five subscales: pain, other symptoms, activities of daily living, sports, and quality of life. All items are scored on a Likert-scale, and each of the five subscales is transformed to a score of 0 (worst) to 100 (best). The original English version has been validated [43], and the Dutch translation is currently being validated in our institution. The EuroQol (EQ-5D) is a validated and extensively used general health questionnaire to measure quality of life [44,45]. It comprises five dimensions: mobility, self-care, usual activities, pain/discomfort and anxiety/depression. Each dimension is marked as either no problems, some problems, or severe problems, which results in a 1-digit number expressing the level selected for that dimension. The digits for five dimensions are combined in a 5-digit number describing the respondent's health state. The numeric rating scale (NRS) for pain consists of an 11-point scale (0 – 10) which represents the whole spectrum of no pain up to the worst pain imaginable [46]. Pain at rest and pain when running will be measured. Patients' satisfaction will be measured using a NRS where 0 indicates no satisfaction and 10 indicates maximally possible satisfaction.

**Figure 2:**
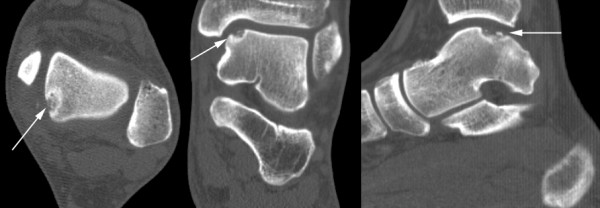
**Preoperative computed tomography (axial, coronal, and sagittal slices) of the left ankle of a 25-year-old female showing a typical osteochondral defect located on the posteromedial talar dome (arrows)**.

To objectively assess bone repair we will obtain multislice helical CT-scans of the affected ankles at baseline and one year after surgery (Figure 2). CT-scanning has been proven to be accurate in the detection and follow-up of ODs of the talus, regarding location and extent as well as healing of the defect [47,48]. The scanning protocol will involve "ultra high resolution" axial slices with an increment of 0.3 mm and a thickness of 0.6 mm, and multi-planar coronal and sagittal reconstructions of 1 mm [49]. The scans will be analyzed twice by a single physician, blinded to both treatment allocation and clinical outcome, measuring completeness, thickness, and level of the subchondral plate (i.e., flush, depressed, or proud) [50]. Additionally, bone volume filling of the defect after one year will be measured, and graded as good (67% to 100%), moderate (34% to 66%), or poor (0% to 33%) [51].

### Adverse events

Any (serious) adverse event during the trial period will be recorded. Adverse events are defined as any undesirable experience occurring to a subject during a clinical trial, whether or not considered related to the investigational treatment, e.g. infection, numbness, or paraesthesia. A serious adverse event (SAE) is any undesirable experience associated with the use of the investigational treatment that results in death, is life threatening (at the time of the event), requires hospitalization or prolongation of existing inpatients' hospitalization, or results in persistent or clinically relevant disability or incapacity. All SAEs will be reported to the central Medical Ethics Committee according to their requirements. Patients suffering from a SAE will stop their PEMF- or sham-treatment.

### Data collection

For each randomized patient a specially designed digital Case Report Form (CRF) will be completed. The CRF consists of a sequential set of instructions with provision for data recording. Internet-based remote data capture will be used for entering, managing and validating data from the investigative sites. For this purpose Oracle Clinical will be used, a program designed to meet industry regulations, including FDA 21CFR Part 11 Rule (March 20, 1997), ICH; Good Clinical Practice: Consolidated Guideline (May 9, 1997) and FDA Guidance for Industry "Computerized Systems Used In Clinical Trials" (May 10, 1999).

All randomized patients are identified by a Patient Identification Number (PIN) in combination with a center number. Trial personnel will not pass names outside the local hospitals. The investigator will ensure that patients' anonymity is maintained. On CRFs or other documents submitted to the coordinating center, patients will not be identified by their names but by a PIN in combination with a center number. The subject identification code list will be safeguarded by the investigator.

### Data acquisition and follow-up

Participating patients will be assessed at the following time points (Table 2):

**Table 2:** Patient assessment.

	Physician									Patient			
	
	Baseline characteristics*	Sport resumption	Work resumption	AAS	AOFAS-AHS	CT	Wound inspection	Compliance	Adverse events	FAOS	EQ-5D	NRS pain	NRS satisfaction
**Preoperative**	X			X	X	X				X	X	X	

**1–2 weeks**							X	X	X				

**1 month**			X					X	X		X	X	X

**2 months**		X	X	X	X		X	X	X	X	X	X	X

**6 months**		X	X	X							X	X	

**1 year**		X	X	X	X	X			X	X	X	X	X

* Baseline characteristics include age, gender, weight, height, affected side, duration of symptoms, type of sport and profession, past medical history, smoking status, and size, localization and classification of osteochondral defect on computed tomography.

AAS = Ankle Activity Score; AOFAS-AHS = American Orthopaedic Foot and Ankle Society – Ankle-Hindfoot Scale; CT = Computed Tomography; FAOS = Foot and Ankle Outcome Score; EQ-5D = EuroQol questionnaire; NRS = Numeric Rating Scale.

1. *Preoperatively*: information letter, informed consent, baseline characteristics (age, gender, weight, height, affected side, duration of symptoms, past medical history, smoking status), type of sport and profession, AAS (2×: before symptoms and at preoperative assessment), AOFAS-AHS, FAOS, EQ-5D, NRS pain (2×: at rest and when running), CT: size, localization and classification of the OD (Table 3) [52]

**Table 3:** Computed tomography classification of osteochondral defects of the talus [52].

Grade	Description
I	Compression
II	Partially fractured but undisplaced
III	Completely fractured but undisplaced
IV	Displaced fracture
V	Radiolucent (fibrous) defect

2. *1–2 weeks postoperatively*: check compliance, (S)AEs, wound inspection (healing, signs of infection)

3. *1 month postoperatively*: check compliance, (S)AEs, resumption of work, EQ-5D, NRS pain (at rest) and satisfaction

4. *2 months postoperatively*: check compliance, (S)AEs, resumption of sport and work, AAS, AOFAS-AHS, FAOS, EQ-5D, NRS pain (at rest and when running, if applicable) and satisfaction, wound inspection, stop PEMF- or sham-treatment

5. *6 months postoperatively*: resumption and maintenance of sport and work, AAS, EQ-5D, NRS pain (at rest and when running)

6. *1 year postoperatively*: resumption and maintenance of sport and work, AAS, AOFAS-AHS, FAOS, EQ-5D, NRS pain (at rest and when running) and satisfaction, (S)AEs, CT: subchondral plate and bone volume filling

### Recording sport resumption

To assess the resumption of sports and work, the patients will keep a diary that will be supplied at inclusion. Every time they perform sports they will record the type of sport and activity level (i.e., professional, competitive, or recreational) in this diary. They will also record the resumption of work, as defined above. This diary will be used for the monitoring of resumption and maintenance of sports and activity levels. At the postoperative visits the patients will be asked to check their diaries and report their sport activities and work resumption, to be filled out on the CRF. At one year the diary will be collected for assessment and confirmation of resumption dates.

### Sample size

Our sample size calculation is based on the combined primary endpoints (a) number of patients that resume and maintain sports during 12 months follow-up, and (b) the time to resumption of sport. Based on our experience it is expected that 50% of patients will resume and maintain sports within one year after the surgical intervention. Offering additional PEMF-treatment, we aim to improve this outcome to 75%. Of the patients who resume to sport the mean time to return to sports after debridement and microfracturing is 15 (standard deviation, 4 weeks) [4]. We consider a 20% reduction in time to return to sports as clinically relevant, i.e., 3 weeks. A sample size of 30 patients in each group (60 patients in total) will have 80% power to detect a joint difference (control group proportion of 0.50 versus treatment proportion of 0.75; control group mean of 15 weeks versus treatment group mean of 12, assuming a common standard deviation of 4), using a Fisher's combination test with a 0.05 two-sided significance level. In reported clinical trials with this device 9% to 13% of included patients dropped out [28,38]. Therefore, 34 patients will be included in each treatment group (68 patients in total).

### Statistical methods

The following baseline characteristics will be summarized using descriptive statistics: number of patients, gender, age, affected side, duration of symptoms (months), prior ankle surgery, body mass index (kg/m^2^), trauma, smoking, size of lesion (mm), classification, duration of PEMF- or sham-treatment (hours), AAS, NRS pain, FAOS, AOFAS-AHS, and EQ-5D. Continuous data will be presented as mean and standard deviation if normally distributed, or as median and range in case of skewed distribution.

The main analysis of this trial consists of a comparison between the treatment groups of the primary outcomes: number of patients who resume and maintain sports and the time to resumption of sport (of the patients who resume sport). The number of patients who resume sport will be analyzed using the two group *X*^2 ^test, whereas the difference in mean weeks to sport resumption will be analyzed by a two group Student's *t*-test. Both *p*-values will be combined using the Fisher's combination test. Kaplan-Meier survival curves and the log-rank test will also be used for comparing time to resumption of sport. The stratification variables will be included in the primary analysis.

The repeated datastructure of the secondary outcomes (AAS, AOFAS-AHS, FAOS, EQ-5D, NRS-pain, and NRS-satisfaction) will be analyzed with linear mixed models, including a time-treatment interaction effect. The number of adverse events and time to resumption of work will be analyzed using a *Χ*^2 ^test or log-rank test, when appropriate. Analyses will be based on the intention-to-treat principle and performed in SPSS. Statistical uncertainty will be quantified via 95% confidence intervals.

### Participating centers and inclusion time

Centers that will participate and their estimated annual inclusions are:

1. Academic Medical Center, Amsterdam, the Netherlands (Prof. Dr. C.N. van Dijk and Dr. G.M.M.J. Kerkhoffs): 24 patients

2. Erasmus MC, University Medical Center Rotterdam, the Netherlands (Dr. D.E. Meuffels and Dr. R. Heijboer): 20 patients

3. Stedelijk Ziekenhuis Roeselare, Belgium (Dr. P.R.N. d'Hooghe): 8 patients

4. Diaconessenhuis, Leiden, the Netherlands (Dr. R. Krips): 8 patients

5. Algemeen Ziekenhuis Sint Lucas, Brugge, Belgium (Dr. G. van Damme): 8 patients

It will take an estimated year to include 68 patients. With one year follow-up, this trial will take an expected two years to be performed.

### Quality assurance

A clinical research associate from our Clinical Research Unit will monitor the trial. All centers will be monitored twice: after the fourth included patient after two months follow-up and after the last patient's last visit. Monitoring will consist of 100% check informed consent procedure, registration of adverse events, completeness of the trial master file, and verification of source data (primary outcome in 10% sample).

### Public disclosure and publication policy

This trial has been registered in the Netherlands Trial Register (NTR1636). Publication will be in accordance with the basic principles of the International Committee of Medical Journal Editors on publication policy [53]. The writing committee will consist of the following people: C.J.A. van Bergen, L. Blankevoort, R.J. de Haan, and C.N. van Dijk. All principal investigators at the participating centers will have the opportunity to scientifically contribute to the manuscript, and, if so, will be listed as an author. If they do not wish to contribute to the manuscript, they will be acknowledged in the order of the number of participants randomized. Other individuals who make substantial contributions to the trial will be acknowledged at the discretion of the writing committee.

## Discussion

This paper describes the rationale and study protocol for conducting a double-blind, randomized controlled trial on the effectiveness of PEMF in the rehabilitation of ankle arthroscopy for ODs of the talus.

The primary outcome measure focuses on sport resumption. This is a difficult measure – as several authors wrote previously [4,54,55] – since a univocal definition does not exist. By clearly defining sport resumption, we aim at providing evidence of any relevant differences between active and passive PEMF-treatment. Moreover, if our definition shows to be useful in the present study, it can be used for the design of future trials.

Regarding the treatment of ODs of the talus, we consider bone regeneration more important than cartilage regeneration. Cartilage is not innervated; the patient's pain probably arises from the bony lesion [56]. Additionally, a differentiation can be made between ODs localized in the ankle joint and those localized in the knee joint. Most studies concerning ODs of the knee focus on cartilage repair rather than bone repair [57,58]. This seems reasonable since the knee joint is less congruent and the ODs are usually localized in high-load-bearing areas. Moreover, the knee joint is more susceptible to osteo-arthritis than the ankle joint [59,60]. The ankle joint, however, has different biomechanical properties. The joint is more congruent and talar articular cartilage is thinner than distal femoral cartilage [61]. The load-bearing contact surface of the ankle joint is somewhat larger [62-64], and the OD is often smaller. Hence, the remaining intact surface of the talar dome is usually sufficiently large to bear the loads; contact surface pressures do not significantly change with talar defects up to 15 mm in diameter [65]. Combining these properties, we believe treatment of ODs in the ankle joint should primarily aim at repair of the subchondral bone, and secondarily at coverage by fibrocartilaginous tissue. In this respect, PEMF-treatment may be particularly suitable for ODs of the talus since its bone-healing capacity has been proven [8,26,27,66].

This trial will contribute to the knowledge of the effectiveness of PEMF, and may improve health care of patients with an OD. Given the modality's relatively simple technology and ease of use, it has high potential to provide a safe and effective additional treatment option for ODs of the talus.

## Competing interests

The authors declare that they have no competing interests.

## Authors' contributions

All authors were involved in the design of the trial. CvB, under supervision of LB and NvD, was responsible for writing this paper, and will act as trial coordinator. RdH was closely involved in the design of the trial and preparation of the manuscript, in particular of the statistical sections. IS assisted in the design of the trial, including the chosen outcome measures. DM, PdH, RK, GvD, and NvD will perform the arthroscopic procedures and will participate in patient inclusion and assessment. All authors have read the manuscript, provided comments on the drafts, and approved the final manuscript.

## Pre-publication history

The pre-publication history for this paper can be accessed here:


